# Retroperitoneal paraganglioma causing proximal ureteric obstruction in a child: diagnostic conundrum and immunohistochemistry characteristics (case report)

**DOI:** 10.11604/pamj.2022.43.79.29159

**Published:** 2022-10-13

**Authors:** Terkaa Atim, Joshua Oluwafemi Aiyekomogbon

**Affiliations:** 1 Department of Surgery, University of Abuja Teaching Hospital and College of Health Sciences, University of Abuja, Abuja, Nigeria,; 2 Department of Radiology, University of Abuja Teaching Hospital and College of Health Sciences, University of Abuja, Nigeria

**Keywords:** Hypertension, extra-adrenal, tumor, resected, case report

## Abstract

Paragangliomas are extremely rare neuroendocrine tumors with an incidence of 0.3 cases per million in children. They arise from several sites in the body including the orbit, ear, nose, larynx, carotid area, mediastinum, duodenum, and the genitourinary tract. Germline mutations have been identified in paragangliomas involving the proto-oncogene RET, tumor suppressor genes VHL, and NF1 The commonest clinical presentation is hypertension but incidentally detected forms have been reported during imaging and on a few occasions the diagnosis was missed. The authors report on a case of a 12-year-old male with a history of fever, left flank pain, vomiting, and headaches, finally diagnosed by pathological examination of a resected retroperitoneal mass invading the proximal left ureter. Immunohistochemistry was positive for neuron-specific enolase, NSE (chief cells), and S-100 protein (sustentacular cells) all consistent with paragangliomas. The challenges faced by the team in the preoperative evaluation and surgical treatment are reported.

## Introduction

Paragangliomas are exceedingly rare neuroendocrine tumors, with a reported incidence of 0.3 cases per million in the pediatric population [1-6]. In 1906, Alezias and Peyron first described extra-adrenal chromaffin tumors and named them paragangliomas, but it was not until 1926 when Mayo reported the first successful excision of paraganglioma [7]. Given the limited experience with paragangliomas in children, we have highlighted some of the dilemmas in diagnosing as well as treating this condition. The World Health Organization (WHO) defines pheochromocytomas as tumors arising from the adrenal chromaffin cells and paragangliomas as tumors derived from extra-adrenal sympathetic chromaffin (sympathetic paraganglioma) and/or parasympathetic tissues (non-chromaffin paraganglioma) of the head and neck [3]. A system of classification for extra-adrenal pheochromocytoma based on location was proposed by Glenner and Grimley in 1974, and they recognized 4 groups namely branchiomeric, intra-vagal, aorto-sympathetic, and viscero-autonomic [7]. Paragangliomas have been reported to arise from several sites in the body including the orbit, ear, nose, larynx, carotid area, mediastinum, duodenum, and the genitourinary tract [4,8-10]. Specific to the genitourinary tract, the bladder constitutes the predominant site (79.2%) whereas the ureter is the least common site (3.2%) [11,12]. Pediatric extra-adrenal paraganglioma occurs most commonly in the retroperitoneum and head and neck, and the diagnosis usually is not suspected at the time of presentation. The commonest presentation in children and adults is hypertension but incidentally detected forms have been reported during imaging and on some occasions, the diagnosis was missed [1-7,11,13,14]. Serum and urinary assays of metanephrines as well as detailed imaging using computerized tomography scans or magnetic resonance imaging are necessary for making the correct diagnosis [13,15]. Germline mutations have been identified in pediatric paragangliomas involving the proto-oncogene RET, tumor suppressor genes VHL and NF1 [5,14]. In low-income settings, patients´ inability to afford extensive and costly investigations coupled with the absence of modern diagnostic facilities pose a challenge in the management of this condition [13,14,16,17]. Surgical resection is the mainstay of treatment for pheochromocytomas and paragangliomas in children with a 15 - 20% 10 year probability of recurrence and up to 20% malignancy rate, hence the emphasis on long-term follow-up [3,5,18]. We present this case of paraganglioma in a 12-year-old African boy to highlight the problems with the overall management, especially in a resource-challenged setting.

## Patient and observation

**Patient information:** we herein present a 12-year-old Nigerian primary school pupil referred to us with a history of fever, left flank pain, vomiting, and headaches. He is the first of two children, a product of normal term delivery, and fully immunized. There was no similar problem in the family. His parents were separated and he lived with his grandmother. He was referred to the pediatricians for control of hypertension after which he had excision of an incidentally detected retroperitoneal paraganglioma involving the proximal left ureter with ureteroureterostomy.

**Figure 1:**
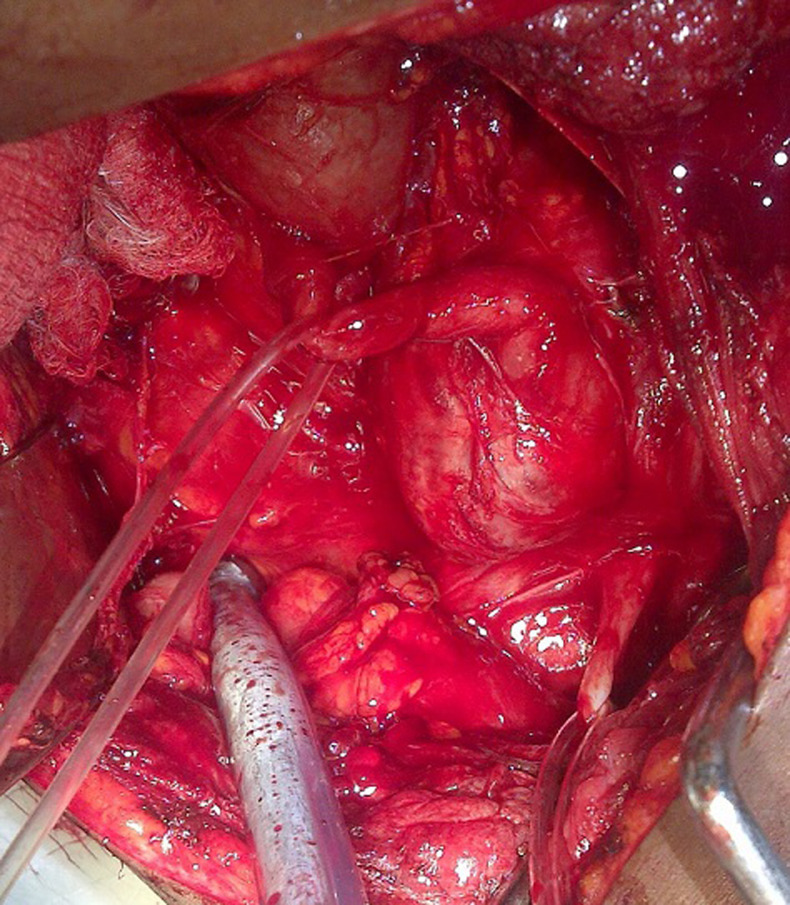
intra-operative picture of retroperitoneal paraganglioma close to the abdominal aorta, encasing the proximal part of the left ureter and causing proximal hydroureteronephrosis

**Clinical findings:** on clinical evaluation, his Blood Pressure was markedly elevated (200/120 mmHg) but no mass was detected on abdominal examination. Serum electrolytes, urea and creatinine, full blood count, and urinalysis were all within normal limits. He commenced higher doses of Lisinopril 10 mg and Aldomet 250 mg with improvement in his blood pressure readings. Abdominal ultrasound revealed left hydrocalicosis and intravenous urography showed prompt excretion of contrast on the right but delayed nephrogram on the left with clubbing and hydronephrosis (Figure 1). A retroperitoneal mass was missed at ultrasonography, and a working diagnosis of left congenital ureteropelvic junction obstruction with hypertension was made. Our center at that time had no facility for either a computed tomography (CT) scan or a magnetic resonance imaging (MRI).

**Timeline:** he was diagnosed with hypertension 2 years before presentation to our facility and initially being managed in a private hospital with no proper diagnosis. He had several hospital admissions and during those episodes, he was treated with oral Lisinopril 5 mg, diazepam 5 mg, and antibiotics. When his symptoms were becoming more frequent and recalcitrant to the treatment, he was referred to us for further evaluation. After stabilization of his blood pressure, laboratory and imaging investigations were carried out and he was worked up to surgery.

**Figure 2:**
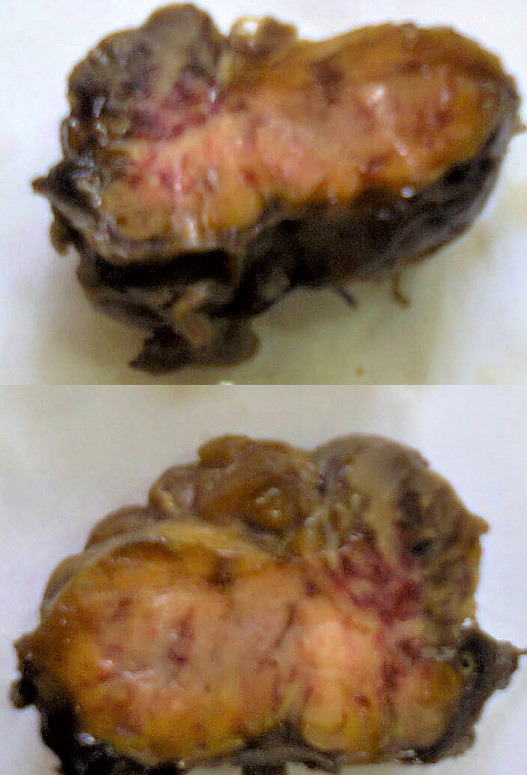
cut section of paraganglioma revealing an irregular greyish brown lobulated soft tissue tumor measuring 8 x 6.5 x 4cm

**Figure 3:**
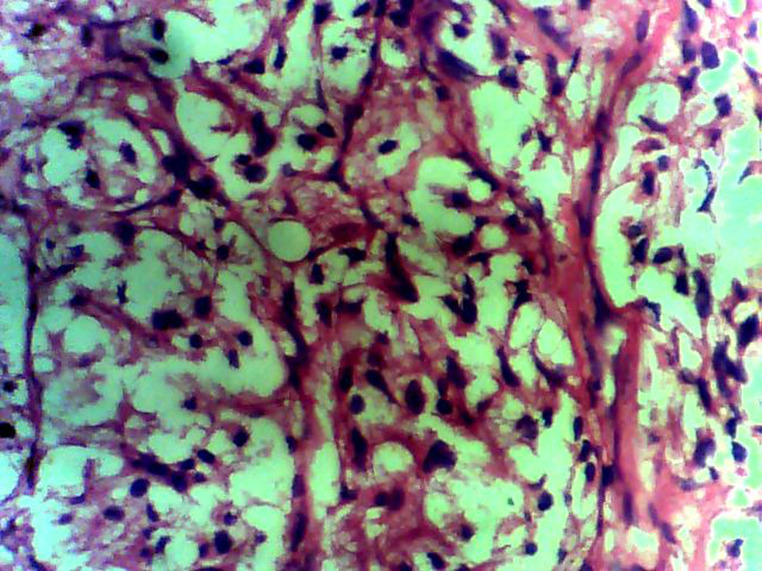
hematoxylin and eosin stain with x 40 magnification showing the paraganglioma depicting the typical cuboidal nests of cells called zellballen, separated by vascularized fibrous stroma showing regular nuclei and few patches of pleomorphism with focal areas of degeneration

**Therapeutic intervention:** during surgery for left pyeloplasty via the flank approach, an incidental finding of a left retroperitoneal mass (paraganglioma) close to the abdominal aorta, encasing the proximal left ureter was discovered and converted to a midline transperitoneal approach which made complete excision of the mass easier (Figure 2, Figure 3). With diligent dissection, fluid monitoring, and intravenous hydralazine titration, the operating team managed to control the blood pressure surges, and the mass was gently mobilized off the wall of the aorta. The mass was excised along with an intervening 4 cm of the left proximal ureter and a primary ureteroureterostomy over a double J stent was done. On the 9th post-operative day, he developed a surgical site infection, which was treated with wound dressing and antibiotics. The ureteric stent, abdominal drain, and urethral catheter were removed sequentially, and secondary wound closure was carried out at 2 weeks. He spent a total of 3 weeks in the hospital.

**Figure 4:**
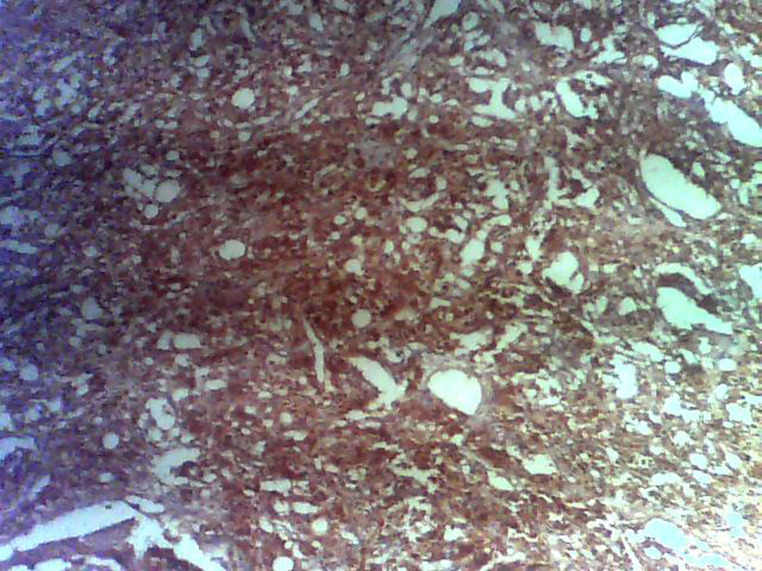
positivity to chromogranin on immunohistochemistry staining confirms the diagnosis of paraganglioma

**Figure 5:**
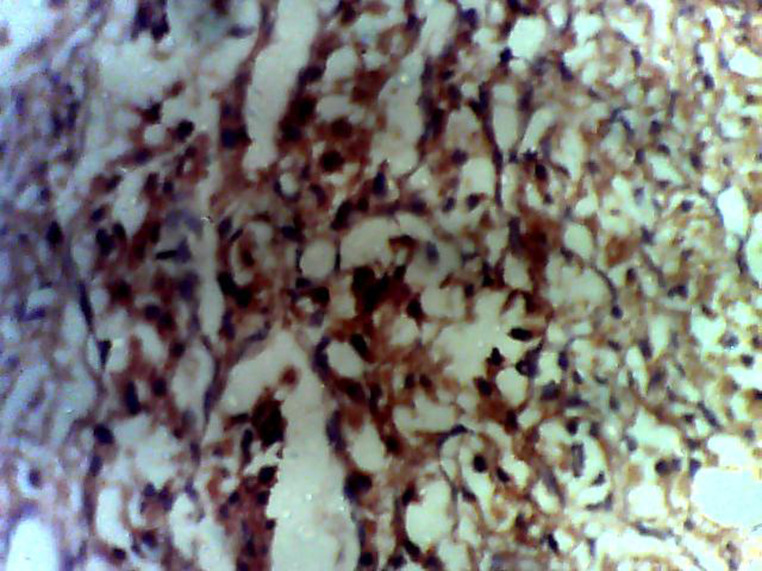
tumour showing positivity for neuron-specific enolase (chief cells) consistent with paraganglioma

**Figure 6:**
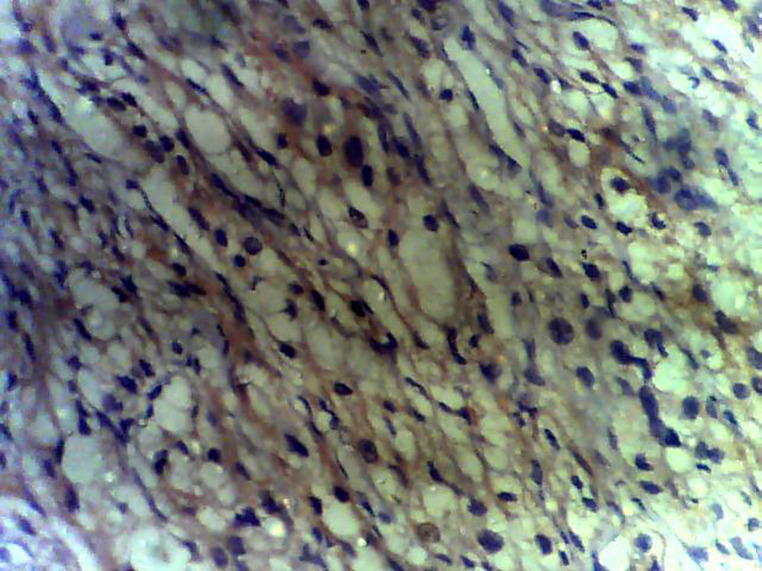
tumour showing positivity for S-100 protein (sustentacular cells) consistent with paraganglioma

**Diagnostic assessment:** surgical pathology showed an irregular greyish brown lobulated soft tissue tumor (paraganglioma) measuring 8x6.5x4cm (Figure 3). It had typical nests of cells intimately related to the thin-walled sinusoidal vessels. The nest of cells was cuboidal “zellballen” separated by vascularised fibrous stroma. The cells showed regular nuclei and few pleomorphic cells with focal areas of degenerative changes (Figure 4). The encapsulated areas of the tumor were intact with fibromuscular tissues from the entrapped parts of the ureter. The excised paraaortic lymph nodes showed reactive lymphoid follicular hyperplasia with features of sinus histiocytosis. There was no evidence of malignancy or metastasis to the lymph nodes. Immunohistochemistry staining of the tumor was negative for cytokeratin (CK7) but positive on the urothelium, and it also showed high Chromogranin positivity (Figure 5). Additionally, the tumor exhibited positivity for neuron-specific enolase NSE (chief cells) and S-100 protein (sustentacular cells) all consistent with the diagnosis of paragangliomas (Figure 6).

**Follow-up and outcomes:** the following tests were done after surgery and the results came out normal: urine VMA 12.1micromol/24hrs (0 - 30.2), urine VMA/Creatinine 2.28 micromol/24hrs (0 - 4.7) urine HVA/24hrs 16 micromol/24hrs (0 - 39) and urine HVA/Creatinine 3 micromol/24hrs (0-7.9). At 4 months follow-up, an intravenous urogram showed prompt bilateral excretion, mild left hydronephrosis, and normal ureters bilaterally. He was followed up for 2 years, initially with no remarkable events.

**Patient perspective:** the informant and caregiver of the patient reported satisfactory care and outcome of the treatment her grandson received during the period of admission and follow-up visits to the urology clinic. He was followed up for two years leading up to this case report.

**Informed consent:** ethical approval was not applicable in this case report. Informed consent was obtained from the caregiver for this publication and any accompanying images.

## Discussion

Paraganglioma in children is a rare catecholamine-secreting tumor that requires a high index of suspicion and supporting diagnostic investigations in making the correct diagnosis [1-3,5,6,8]. Although most paragangliomas occurring below the diaphragm are functional, thus producing symptoms due to the excessive secretion of catecholamines, a few have been reported to be non-functional [5,7,11]. The usual presentation of pediatric patients with paragangliomas is similar to adults and may include; headache, palpitation, sweating, and hypertension, as well as symptoms from tumor compression of nearby structures [2,7,19]. The need to thoroughly investigate children with hypertension for paraganglioma/pheochromocytoma cannot be overemphasized, bearing in mind other common causes such as coarctation of the aorta and renovascular hypertension [5].

This case presents a possible lack of perception and challenges posed to clinicians in the management of paragangliomas in children, especially in low-income settings. The index patient had hypertension secondary to an incidentally detected retroperitoneal paraganglioma at surgery causing left ureteropelvic junction obstruction. de Waard D *et al*. in their study noted that hypertension should be sought in patients with dilated or obstructed upper urinary tract and considered this as an indication for surgery and relief of obstruction cures hypertension in the greater majority of patients [20,21]. In instances where retroperitoneal paragangliomas produce compression symptoms or invade adjacent organs, they could be misdiagnosed as tumors of the affected structures, just as in this index case [22]. Pain from paragangliomas could occur when the tumor compresses adjacent structures [2,6,7,23-25]. In resource-challenged settings where a lack of comprehensive laboratory and imaging investigations is not infrequent, this rare disease could be easily missed with grave consequences [16,17,26,27].

The average age of onset of paragangliomas in children is 11 years with a male to female ratio of 2: 1 and this is comparable to the index case [4]. Similar to our earlier review on congenital ureteropelvic junction obstruction, few other researchers also documented ureteric obstruction from retroperitoneal tumors involving the genitourinary tract [11,12,19]. It is clear enough that retroperitoneal extra-luminal masses could lead to ureteric obstruction and in a review on ureteropelvic junction obstruction in their institution, Atim *et al*. observed that flank pain is a common symptom in these cases [7,21]. They equally opined that in their setting, ultrasound and intravenous urogram were widely used in the evaluation of flank pain and hydronephrosis [21]. Ultrasonography and intravenous urography, however, missed the retroperitoneal paraganglioma in this index case and reported only left hydronephrosis.

Among the indications for surgical intervention in children with hydronephrosis are hypertension, flank pain, renal impairment, infection, and stones, the initial two features being apparent in our patient [4,5,20,21,23]. A thorough workup including an assay of serum or urine catecholamines and their metabolites is paramount in the evaluation of hypertension in children. Unfortunately, this investigation could not be done on our patient before surgery due to the high cost of the tests and the lack of reagents in our center at the time. Similar circumstances have been alluded to by two groups of researchers Chukwu *et al*. and Abdelkhalek *et al*. as being partly responsible for delay in diagnosis and oftentimes misdiagnosis in resource-poor settings [16,17]. In addition, a failure on the part of clinicians to recognize signs and symptoms suggestive of this rare condition may also be responsible for misdiagnosis [26]. Two weeks following surgical excision, the VMA and serum catecholamines were all normal in the patient. Surgical excision remains the treatment of choice for paragangliomas to prevent severe end-organ complications and fatal outcomes in children [5,18]. To further characterize the nature of the excised tumor we relied on histopathological and immunohistochemistry analysis.

The absence of cytokeratin expression by paragangliomas at immunohistochemistry has been used by pathologists to separate them from other neuroendocrine tumors [28]. Immunohistochemistry analysis in the index patient revealed a positive reaction to Vimentin, chromogranin, and synaptophysin (Figure 5) confirming the diagnosis of paraganglioma [11]. The suspicion of paragangliomas should in children be considered after excluding more usual causes of hypertension such as cardiovascular or renovascular diseases, renal parenchymal disease, renal congenital anomalies, thyroid disease, Cushing syndrome, and congenital adrenal hyperplasia [4].

## Conclusion

The occurrence of hypertension in the pediatric age group should arouse a need to screen for pheochromocytoma-paraganglioma syndrome. Early treatment is crucial to avoid life-threatening cardiovascular complications. This case highlights the significance of a high index of suspicion and detailed clinical evaluation in identifying these patients and offering them timely treatment. The importance of entertaining a broad differential diagnosis and carrying out thorough laboratory and imaging investigations hold the key to correct diagnosis and successful management. The mainstay of treatment remains surgery, and the outcome is good following gross complete resection. To the best of our knowledge, this is one of the few reports of surgical management of paragangliomas in children and the first detailed report of the immunohistochemistry characteristics of the tumor in our environment.
